# Medicaid expenditures for children living with smokers

**DOI:** 10.1186/1472-6963-11-125

**Published:** 2011-05-25

**Authors:** Douglas E Levy, Nancy A Rigotti, Jonathan P Winickoff

**Affiliations:** 1 Mongan Institute for Health Policy, Massachusetts General Hospital, Boston, Massachusetts, USA; 2 Tobacco Research and Treatment Center, General Medicine Division, Massachusetts General Hospital, Boston, Massachusetts, USA; 3 Department of Medicine, Harvard Medical School, Boston, Massachusetts, USA; 4 MGH Center for Child and Adolescent Health Policy, General Pediatrics Division, MassGeneral Hospital for Children, Boston, Massachusetts, USA; 5 Department of Pediatrics, Harvard Medical School, Boston, Massachusetts, USA

## Abstract

**Background:**

Children's exposure to secondhand smoke is associated with increased morbidity. We estimated Medicaid expenditures for children living with smokers compared to those living with no smokers in the United States.

**Methods:**

Data were overall and service-specific (i.e., inpatient, ambulatory, emergency department, prescription drug, and dental) annual Medicaid expenditures for children 0-11 years old from the 2000-2007 Medical Expenditures Panel Surveys. Smokers' presence in households was determined by adult respondents' self reports. There were 25,835 person-years of observation. We used multivariate analyses to adjust for child, parent, and geographic characteristics.

**Results:**

Children with Medicaid expenditures were nearly twice as likely to live with a smoker as other children in the U.S. population. Adjusted analyses revealed no detectable differences in children's overall Medicaid expenditures by presence of smokers in the household. Medicaid children who lived with smokers on average had $10 (95% CI $3, $18) higher emergency department expenditures per year than those living with no smokers.

**Conclusions:**

Living with at least one smoker (a proxy for secondhand smoke exposure) is unrelated to children's overall short-term Medicaid expenditures, but has a modest impact on emergency department expenditures. Additional research is necessary to understand the relationship between secondhand smoke exposure and long-term health and economic outcomes.

## Background

One in three children in the U.S. lives with a smoker,[1] and extensive research has established the detrimental effect of secondhand smoke exposure (SHS) on children's health [2]. Because children spend much of their time in the home, outside the sphere of most clean air regulation, they have higher levels and rates of exposure to SHS than adults [3]. Short-term consequences of pediatric exposure to household SHS include increased risk for asthma, bronchitis, bronchiolitis, sinusitis, middle ear infections, and sudden infant death syndrome [2,4-7]. Longer-term sequelae include reduced lung function and development, and cognitive impairment with specific deficits in reading, math, and visuospacial reasoning [6,8-12].

Though the adverse health outcomes children face due to SHS exposure are of paramount importance, understanding the relationship between SHS exposure and children's health expenditures is essential to prioritizing scarce health care resources and motivating policy makers to take corrective action. There have been studies estimating the health care costs of children's SHS exposure [13-17]. However, absent from the existing literature is an examination of children's SHS exposure and its relationship to expenditures in the public health insurance programs that cover children in lower income families in the United States: Medicaid and the Children's Health Insurance Program (CHIP). Nearly one third of children in the United States were covered by Medicaid or CHIP in 2005 [18] and low-income children are much more likely to live with a smoker [1]. Thus, the impact of SHS exposure is concentrated in this population. In addition, by focusing on children who (within states) all have the same health insurance, we will remove some of the heterogeneity in ability and propensity to access medical care that may have confounded earlier studies [14]. In the current study, we use the Medical Expenditures Panel Survey (MEPS) to examine the relationship between children's Medicaid/CHIP expenditures and living with a smoker.

## Methods

### Data

Our sample consists of children 0-11 years old who were included in the 2000-2007 rounds of the MEPS, a national survey representative of the non-institutionalized population of the United States. The MEPS is funded by the Agency for Healthcare Research and Quality (AHRQ) and conducted by in-person interview. We focus on children less than 12 years old to reduce the likelihood that a child's tobacco smoke exposure is due to the child's own tobacco use.

Households participating in the MEPS complete five interviews over a two year period and annual response rates ranged from 57-66% over the study time period [19]. Detailed information on demographics, socioeconomic status, health status, health care utilization, insurance coverage, and health expenditures was collected for each household member. Survey staff obtain expenditure data by contacting the providers identified by respondents and requesting information on payments received for services/products provided to the respondent [20]. We included only children who were enrolled in Medicaid for some part of the year (here and henceforth, we use the term Medicaid to indicate Medicaid and CHIP combined). Our analyses were conducted with the person-year as the unit of observation, so our findings relate to average annual medical expenditures per child.

#### Dependent Variables

Average annual expenditures for children living with smokers and those not living with smokers were calculated. Because we were primarily interested in the effect of SHS exposure on expenditures to public programs, our primary analyses include only expenditures paid for by Medicaid. Costs borne by other payers, including families' out-of-pocket payments (copayments, coinsurance, deductibles, uninsured care) were excluded. As a sensitivity analysis, we also assessed Medicaid recipients' total health expenditures (not just those paid by Medicaid) as a function of living with a smoker. Multivariate regression models were used to better isolate the effect of living with a smoker on expenditures.

Dollar amounts reflect what providers report was actually paid for medical care, not the price they charged for the care. Expenditures for care delivered under capitated payment arrangements were imputed by AHRQ [21]. We considered both overall expenditures (exclusive of well-child care) and expenditures for specific medical services. Specific services included inpatient care, emergency department care, ambulatory care (exclusive of well-child care), well-child care, dental care, and prescription pharmaceuticals. In all cases, we exclude expenditures for vision care and chiropractic care which should not be causally affected by SHS exposure. We adjust expenditures for inflation to 2007 dollars according to the methods recommended by AHRQ [22].

#### Independent Variables

The principal independent variable of interest was exposure to SHS. The MEPS does not include any direct measures of children's exposure to SHS, for example serum cotinine levels, so we created a proxy for exposure by determining whether or not there were any smokers in the child's home according to adult respondents' self-reports of smoking ascertained at the midpoint of the survey year. Evidence suggests that living with a smoker is a sensible proxy for SHS exposure, particularly for younger children who spend a greater proportion of their time at home, given the evidence that living with a smoker increases exposure to tobacco smoke. For children, the home is "the dominant site of exposure" [2]. Only 27% of self-reported smokers have strict home smoking bans, while 88% of non-smokers have such a ban [23]. Children living with smokers in homes where there is no home smoking ban have the highest levels of SHS exposure, but even children living with smokers in homes where there is a no smoking rule have SHS exposures that are significantly greater than children who live in households with no smokers at all [24,25]. As a sensitivity analysis, we also tested whether there was a differential effect according to the number of smokers in the home.

In multivariate analyses, we controlled for a number of potential confounders. We controlled for the child's age, sex, race, and ethnicity. We controlled for family characteristics including mother's age, presence of both parents in the home, household poverty status, and highest education achieved by either parent. Except where well-child care is the dependent variable, we controlled for age-adjusted number of well-child visits. Finally, we controlled for urban/rural location and Census region.

### Statistics

Bivariate differences in the characteristics of children living with or without smokers were assessed using Pearson chi squared statistics. For our regression analyses, we calculated two-part and aggregate models. In the two-part model, part one calculated the probability of any (i.e., non-zero) expenditures using logistic regression. Part two calculated the level of expenditures *conditional *on having any expenditures using a generalized linear model (GLM). In the aggregate models, we calculated the level of expenditures for all children, *not *just those with non-zero expenditures, using a GLM. Following current statistical methodology, we assessed a number of modeling strategies and determined that a GLM using a log link and following a Poisson distribution fit the data best [26,27]. Regression-adjusted mean expenditures and their differences were calculated only for children living with smokers as this is the group that would be affected by changes in household smoking. Using the regression model, we predicted expenditures under the observed scenario that a child lived with smokers and under a counterfactual scenario where the same child did not live with smokers. Mean adjusted annual expenditures in each scenario were calculated as the average of the predicted values. Confidence intervals for the difference across scenarios were calculated by predicting expenditures using the lower and upper limits of the 95% confidence interval for the regression coefficient on the presence of smokers in the home. All statistics were estimated with Stata 10.1 (Stata Corporation, College Station, TX) using balanced repeated replicates to account for the MEPS design characteristics and repeated observations within individuals. The analyses took place in 2010 and 2011.

Because the study involved only publicly available, deidentified data, it was deemed exempt from review by the Massachusetts General Hospital Institutional Review Board.

## Results

### Sample Characteristics

There were 16,154 unique Medicaid beneficiaries 0-11 years old and 25,835 person-years of observation for the 2000-2007 rounds of the MEPS, representing over 17 million children per year. The average child was enrolled in Medicaid for 10 months of the year, and 65% were enrolled for all 12 months. There was no difference in the duration of Medicaid enrollment between children living with smokers and children with no smokers in the home.

**Figure 1:**
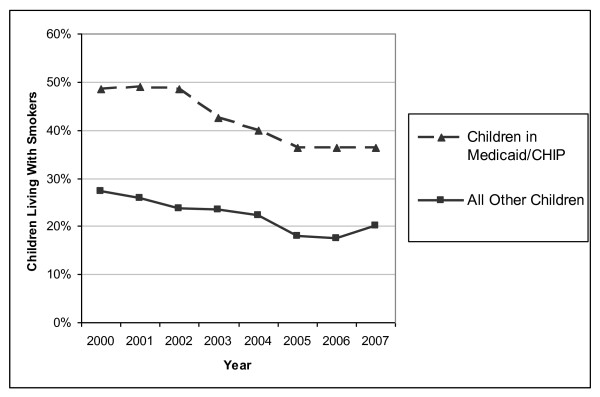
**Trends in the proportion of children living with smokers**.

While 49% of the children in this population lived with at least one smoker in 2000, that number had declined to 36% by 2007 (Figure 1). Levels of cohabitation with smokers were substantially higher in Medicaid children than among non-Medicaid children, where levels declined from 27% in 2000 to 20% in 2007. On average, over our study period Medicaid children under age 12 were 91% more likely to live with a smoker than similarly aged children outside the Medicaid program. Across all study years, 56% of households had no smokers, 30% had one smoker, 12% had two smokers, and 2% had 3 or more smokers. Among study households with at least one smoker, 56% had a mother who smoked, 39% had a father who smoked, and 35% had smokers other than one of the child's parents.

There were statistically significant differences on most respondent characteristics between children living with at least one smoker and those who did not live with a smoker (Table 1). Among the sociodemographic characteristics, children living with smokers were somewhat more likely to be white, less likely to be black, and much less likely to be Hispanic. Mirroring differences observed in most populations of smokers, parents in households with smokers were less educated and had lower incomes, even within this Medicaid population. Children living with smokers were less likely to have both parents in the household. They were also more likely to live in urban areas, more likely to live in the Midwest, and less likely to live in the West.

**Table 1:** Characteristics of the study population.

*Characteristic*		*0 Smokers in Home*	*≥1 Smoker in Home*	*p-value*
**Child**				

Age	0-5	53%	55%	.23

	6-11	47	45	

Race	White	66	70	<.001

	Black	27	22	

	Other	6	7	

Ethnicity	Hispanic	40	20	<.001

Well-child care	<75^th ^percentile	64	65	.90

**Family**				

Highest Parent Education	<HS	9	6	<.001

	Some HS	20	25	

	HS	38	42	

	Some college	25	22	

	≥ College	8	5	

% Fed. Poverty Level	<100%	42	48	<.001

	100-399%	56	49	

	>400%	3	3	

Both parents in household		55	49	<.001

Urban		82	77	<.001

Census Region	Northeast	15	17	<.001

	South	41	40	

	Midwest	14	24	

	West	30	19	

Mother's age	<25	23	31	<.001

	25-34	50	46	

	35-44	23	20	

	≥45	4	3	

### Medicaid Expenditures

Table 2 presents predicted expenditures, based on our regression models, for an average child who does or does not have a smoker in the house, comparing our estimates from the unadjusted model to the model where we adjust for potential confounders. In nearly all cases, point estimates suggest that living with a smoker is associated with higher Medicaid expenditures, though few results were statistically significant. In the unadjusted analysis, we estimated that living with a smoker was associated with an additional $213 in overall Medicaid expenditures (exclusive of well-child care) per child per year, but the difference was not statistically significant (95% CI -$56, $580). However we found that living with a smoker was associated with significantly higher emergency department ($13 per child per year, 95% CI $6, $22) and prescription drug expenditures ($51 per child per year, 95% CI $8, $111). An apparent difference in dental expenditures was not statistically significant.

**Table 2:** Estimated annual Medicaid expenditures by presence of smoker in home (2007$)

	Unadjusted			**Adjusted **^**a**^		
	*Smoker in House*		*Difference*	*Smoker in House*		***Difference***^***b***^
	No	Yes	*(95% CI)*	No	Yes	*(95% CI)*
*Overall Expenditures*^*c*^	*$799*	*$1012*	*$213 (-56, 580)*	*$862*	*$1012*	*$149 (-68, 427)*

Inpatient	360	450	90 (-66, 328)	380	450	70 (-74, 283)

Emergency Dept.	45	58	13 (6, 22)***	47	58	10 (3, 18)**

Ambulatory^*c*^	226	246	20 (-21, 69)	248	246	3 (-41, 56)

Prescription	98	148	51 (8, 111)*	113	148	35 (-3, 87)

Dental	54	71	17 (-2, 44)	58	71	13 (-4, 35)

Well-child	77	73	-4 (-12, 6)	81	73	-8 (-15, 1)

^a ^Adjusts for child's sex, race, and ethnicity, age-adjusted well child visits (except where well-child visits are the outcome), urban/rural location, Census region, mother's age, presence of both parents in the home, household poverty status, and highest education achieved by either parent.

^b ^Estimates of the difference in predicted expenditures for children observed to be living with smokers when smokers are or are not present. The 95% confidence interval is based on the confidence interval for the coefficient on having a smoker in the house.

^*c *^Excludes well-child care, chiropractic, and vision care.

*p < 0.05; **p < 0.01; ***p < 0.001

Adjusting for potential confounders generally attenuated the point estimates for the relationship between Medicaid expenditures and living with a smoker, sometimes substantially. After adjusting for potential confounders, only emergency department expenditures remained significantly associated with living with a smoker, a difference of $10 per child per year (95% CI $3, $18). Apparent differences for prescription drug and dental expenditures were not statistically significant. There was a strong indication that expenditures for well-child care were *lower *among children living with smokers, but this estimate was also not statistically significant (-$8 per child per year, 95% CI -$15, $1).

To further probe the relationship between household smoking and Medicaid expenditures, we present results from the two-part and aggregate models of expenditures in Table 3. For overall expenditures, there was no relationship between the presence of smoking in the home and overall Medicaid expenditures for either the two-part or aggregate models. Because inpatient and emergency expenditures are so rare, their two-part models did not converge and we were not able to separately assess the two components contributing to aggregate expenditures for the services. For prescription drug expenditures, the two-part model highlighted a significant increase in the likelihood of *any *expenditures among children living with smokers. However, this slight increase did not carry through to the conditional or aggregate models. On the other hand, for dental expenditures, there was no difference in the likelihood of *any *dental expenditures, but there was a significant increase in conditional expenditures for children living with smokers. Again, this did not carry through to the aggregate model. There was no evidence that ambulatory care expenditures exclusive of well-child care were related to the number of smokers in the home. The trend towards lower well-child expenditures among children living with smokers was driven almost entirely by lower medical expenditures among those who had non-zero expenditures.

**Table 3:** Adjusted^*a *^two-part and aggregate models of expenditures as a function of presence of smoker in home

	Two-part model		Aggregate model
	***Part 1: odds ratio for any expenditures (95% CI)***	***Part 2: conditional expenditure; GLM coefficient (95% CI)***	***Overall expenditure: GLM coefficient (95% CI)***

*Overall Expenditures*^*b*^	*1.09 (0.99, 1.22)*	*0.14 (-0.13, 0.40)*	*0.16 (-0.08, 0.40)*

Inpatient	DNC	DNC	0.17 (-0.22, 0.56)

Emergency Dept.	DNC	DNC	0.20 (0.07, 0.33)**

Ambulatory^*b*^	1.03 (0.95, 1.13)	-0.01 (-0.19, 0.18)	0.01 (-0.18, 0.20)

Prescription	1.11 (1.02, 1.21)*	0.19 (-0.10, 0.48)	0.27 (-0.02, 0.57)

Dental	0.95 (0.82, 1.09)	0.20 (0.04, 0.35)*	0.20 (-0.07, 0.47)

Well-child	0.98 (0.88, 1.09)	-0.08 (-0.17, 0.01)	-0.10 (-0.21, 0.01)

DNC: model did not converge

^*a *^Adjusts for child's sex, race, and ethnicity, age-adjusted well child visits (except where well-child visits are the outcome), urban/rural location, Census region, mother's age, presence of both parents in the home, household poverty status, and highest education achieved by either parent.

^*b *^Excludes well-child care, chiropractic, and vision care.

*p < 0.05; **p < 0.01

### Sensitivity Analyses

We estimated models of total health expenditures (regardless of payer) as a function of living with a smoker for overall expenditures and for each separate service (inpatient, emergency, etc.). The statistical significance and relative differences observed for total health expenditures mirror those of Medicaid-paid expenditures, and in some cases (for example, emergency department expenditures) exhibited even smaller differences. Because our findings for total health expenditures are similar to those described above, we do not report them separately.

We also estimated models where smoking status was defined by the number of smokers in the household. Because only 2% of households had 3 or more smokers, we defined household smoking as having 0 smokers, 1 smoker, or 2 or more smokers. We estimated models that assumed a linear relationship with respect to the number of smokers, and models where the three categories of household smokers (0, 1, or ≥2) were included as indicator variables, which would not assume a linear relationship. Both of these models also closely mirrored the findings reported in Tables 2 and 3, so we do not report them separately. Model coefficients indicated higher expenditures for 2 or more smokers compared to one smoker for overall expenditures, inpatient expenditures, emergency department expenditures, and prescription drug expenditures, but none of the estimates were statistically significant.

## Discussion

Using nationally representative survey data, we present the first study examining the relationship between children's Medicaid expenditures and the presence of a smoker in the home. We confirmed that children under 12 and covered by Medicaid were nearly twice as likely to live with a smoker as other children in this age group. Confounding characteristics explained a substantial portion of the difference in medical expenditures between Medicaid children living with or without smokers, and there was no evidence that household smoking was related to children's overall Medicaid expenditures. This is surprising, given the substantial evidence linking SHS exposure to poor child health. We posit that the lack of an observed relationship between household smoking status and health care expenditures may be because the childhood illnesses caused or exacerbated by SHS exposure are not common enough and/or severe enough to raise average medical expenditures. We did see higher emergency department expenditures among children living with smokers, possibly pointing to more/more severe asthma among exposed children. Our estimates of emergency department expenditures included controls for age-adjusted well-child visits, and we found no relationship between non-well-child ambulatory care expenditures and living with a smoker; thus it seems unlikely that the observed increase in emergency department spending simply reflects a substitution from ambulatory to emergency care. We also found that children living with smokers had a higher likelihood of non-zero pharmaceutical costs, potentially due to medications prescribed to treat respiratory illnesses. However, neither of these factors was enough to result in significantly higher overall Medicaid expenditures.

Previous studies in the literature yielded mixed results with respect to the relationship between household smoke exposure and children's medical expenditures. Two of the earliest studies found substantial relationships between household smoking and children's medical expenditures. Aligne and Stoddard estimated that exposure to SHS raises expenditures on children's health care by $203 to $284 per year in 2007 dollars [13]. Their method used a literature review employing separately measured health outcomes and costs, which assumed all adverse health events were treated and that children's healthcare costs for a given condition were identical whether that condition was caused by SHS exposure or not. If these assumptions are invalid, particularly the former, that would bias their findings towards a higher cost of SHS exposure. In addition, at the time of their study (using data prior to 1997), they estimate that more than 50% of children lived with at least one smoker, a figure that is substantially higher than what we observe for children overall. Stoddard and Gray focused on expenditures for respiratory illnesses in children under the age of five who lived with a mother who smoked [17]. Using the 1987 National Medical Expenditures Survey (NMES), the authors found annual expenditures of $156 to $228 per exposed child in 2007 dollars. However, the NMES only has data on what providers *charged *for medical care, not what they were paid, which is typically much lower. Therefore, NMES data likely overestimate the relationship between SHS exposure and expenditures. A more recent study by Hill and Liang using the MEPS found children 0-4 years old living where smoking took place in the home had $128 per year (2007 dollars) higher expenditures for respiratory conditions than children who had no smoking in the home [15]. As the authors suggest, because the youngest children spend the most time in the home, their exposure levels are likely higher that what would be seen in older children.

Other studies have found no relationship between household smoking and children's health expenditures. McBride et al studied children whose parents were participating in smoking cessation trials in a managed care setting, finding no evidence of increased expenditures for children living with smokers [16]. The study most similar to ours was published by Florence et al [14]. Using the 2000-2003 MEPS, they were also unable to detect any relationship between children's expenditures and living with a smoker when examining all-payer spending for children 0-11 years old. Finding a lower odds of non-zero medical expenditures among children living in households with smokers, the authors posited that unobserved caregiver characteristics (possibly parents' inclination to seek medical care), correlated with both the presence of smokers in the household and children's medical expenditures, might explain the lack of an overall relationship between household smoking and children's medical expenditures. Such an explanation does not appear plausible in our analysis of Medicaid recipients. We find no evidence that children living with smokers have a lower likelihood of having non-zero expenditures than children who do not live with smokers. By restricting our sample to Medicaid recipients, we have a group that is likely more homogeneous on observed and unobserved characteristics, particularly the ability and propensity to access medical care, than the sample studied in Florence et al.'s analysis. While they control for income, education, and insurance coverage, there may be relationships between those variables and the presence of smokers in the household that could not be captured in their model.

Even within our sample of Medicaid recipients, the relationship between children's health expenditures and living with a smoker is highly complex, as evidenced by significant differences in demographic, socioeconomic, and geographic characteristics between children living with no smokers and those living with at least one. Controlling for these factors attenuated the relationship between our exposure measure and medical expenditures, suggesting they are important confounders.

Our analysis should be considered in the context of certain limitations. Most central to our analysis, we did not directly measure exposure to SHS. Nevertheless, we have argued that living with a smoker is a reasonable proxy measure. We focused entirely on the short-term differences in children's expenditures according to whether or not they lived with a smoker. The present study does not address the question of how childhood exposure to SHS affects healthcare spending over the life course. Nor does it consider the effects of *in utero *exposure, long-term health effects, or potential increases in the likelihood that the child him-/herself eventually becomes a smoker due to the establishment of social norms in the home indicating smoking is acceptable behavior [28]. We also did not examine the extent to which smoking may have contributed to the socioeconomic situation of the family, with the average pack-a-day smoker spending $1800 each year on cigarettes [29]. We focused entirely on expenditures in the context of Medicaid, which are substantially different from the private insurance setting. Lastly, any effect of childhood SHS exposure on cognitive impairment and learning difficulties would lead to SHS-attributable costs that fall outside the health sphere [30].

## Conclusion

The present study indicates that living with at least one smoker, a proxy for increased levels of SHS exposure, is unrelated to children's overall Medicaid expenditures, but may have a modest impact on emergency department expenditures. While reducing Medicaid expenditures does not appear to be a rationale for reducing the number of children living with smokers, the most powerful argument for reducing children's exposure to the toxins in SHS remains reducing childhood morbidity. Additional research is necessary to understand the relationship between secondhand smoke exposure and long-term health and economic outcomes.

## List of Abbreviations

SHS: Secondhand smoke; CHIP: Children's Health Insurance Program; MEPS: Medical Expenditures Panel Survey; AHRQ: Agency for Healthcare Research and Quality; NMES: National Medical Expenditures Survey

## Competing interests

The authors declare that they have no competing interests.

## Authors' contributions

DEL conceived and designed the study; acquired, processed, and analyzed the data; and drafted the manuscript. NAR and JPW participated in the interpretation of the analyses and helped draft the manuscript. All authors read and approved the final manuscript.

## Pre-publication history

The pre-publication history for this paper can be accessed here:

http://www.biomedcentral.com/1472-6963/11/125/prepub
